# Age, Dental State and General Health as Risk Factors for Xerostomia and Hyposalivation

**DOI:** 10.1155/ijod/3062701

**Published:** 2026-10-06

**Authors:** Foteini Spyraki, Philippe Mojon, Najla Chebib, Frauke Müller, Sabrina Maniewicz

**Affiliations:** ^1^ Division of Gerodontology and Removable Prosthodontics University Clinics of Dental Medicine University of Geneva Geneva Switzerland unige.ch; ^2^ Department of Rehabilitation and Geriatrics University Hospital of Geneva Geneva Switzerland hug-ge.ch; ^3^ Center of Excellence in Precision Medicine and Digital Health Department of Physiology Faculty of Dentistry Chulalongkorn University Bangkok Thailand chula.ac.th

**Keywords:** aging, geriatric patients, hyposalivation, risk factors, saliva, salivary glands, xerostomia, xerostomia inventory

## Abstract

**Introduction:**

Xerostomia and hyposalivation are common among adults and may significantly influence both oral health and overall quality of life.

**Objective:**

The aim of this study was to analyze patient‐related risk factors of xerostomia and hyposalivation in a clinical cohort.

**Materials and Methods:**

The clinical evaluation included measurements of unstimulated and stimulated salivary flow rates. Furthermore, the xerostomia inventory (XI) was employed, and an additional general question on xerostomia was included. For analyzing associations, nonparametric tests, chi‐square tests, correlation analyses, and binary logistic regression were used.

**Results:**

A total of 116 adult volunteers participated in this study (59.5% women; mean age: 50.2 ± 18.7 years). Xerostomia was found in 14.7% of participants, while hyposalivation at rest and under stimulation was detected in 21.6% and 4.3%, respectively. The participants with xerostomia were significantly older (*p* = 0.002), and had fewer remaining teeth (*p* = 0.03). Hyposalivation at rest showed significant correlations with older age, higher number of medications, fewer remaining teeth, lower stimulated salivary flow rate, and higher XI scores (all *p*  < 0.05). Age was the only independent predictor of hyposalivation at rest, while hyposalivation at rest was the strongest predictor of xerostomia in binary logistic regression analysis.

**Conclusion:**

Age, hyposalivation at rest, and XI score are the strongest markers of reduced salivary flow and xerostomia.

## 1. Introduction

Xerostomia and hyposalivation are both closely related but distinct medical conditions that can have an impact on the overall quality of life, especially in older adults [1, 2]. Xerostomia is the subjective feeling of a dry mouth, while hyposalivation is the objectively measured decrease in salivary flow rate [3]. Patients may experience oral dryness with or without a decrease in saliva secretion, while others may have decreased salivary flow asymptomatic. Hyposalivation has been documented in nearly 30% of the general adult population presenting with xerostomia, emphasizing the importance of making a distinction between the two conditions [4]. Xerostomia prevalence is reported at between 0.9% and 64.8% of adults, with higher rates being described in older adults [5]. Hyposalivation, however, has been identified in 33% of older adults and is frequently associated with polypharmacy, radiotherapy, or systemic diseases [6].

The clinical effects of xerostomia and hyposalivation are not limited to a feeling of discomfort; their effects can significantly affect both oral and systemic health [4]. Saliva is vital in the regulation of oral homeostasis, pH balance, digestion, and protection against infections [7, 8]. A diminished salivary flow rate can cause mastication, deglutition, and speech difficulty, affecting a person’s quality of life considerably [9, 10]. From a dental standpoint, hyposalivation is closely correlated with the prevalence of dental caries, oral candidiasis, and burning mouth syndrome [11]. Lower saliva can also lead to demineralization of teeth, which causes hypersensitivity and a greater risk of erosion [12]. Dry mouth has also been implicated in changes in taste, halitosis, and increased susceptibility to denture discomfort as the mucosal membranes tend to be more sensitive [9].

Identifying the risk factors for xerostomia and hyposalivation has an important role in the optimization of effective prevention and management strategies [13]. The prevalence of these conditions is expected to rise as a result of longer life expectancy, the increasing population with chronic diseases, and the intake of numerous medications, particularly among the elderly [14]. The identification of nonmodifiable and modifiable risk factors allows clinicians to develop strategies for high‐risk persons. Current treatment strategies mainly center on symptom relief, with interventions like using saliva substitutes, sialagogues, and lifestyle modification [15–17]. However, addressing patient‐related risk factors can lead to a more comprehensive and long‐lasting solution, facilitating a better understanding and suggesting evidence‐based standards of diagnosis, prevention, and treatment. Thus, the aim of the current study is therefore to identify risk factors for the development of xerostomia and hyposalivation by analyzing the relevant demographic, medical, and behavioral factors associated with these two conditions.

## 2. Materials and Methods

The study received ethical approval from the local ethics committee (CCER 2021‐01432). The study sample consisted of a convenience sample of adult patients recruited from the patient pool of the University Clinics of Dental Medicine of the University of Geneva, Switzerland. Participants were required to be at least 18 years old and to provide written informed consent. Exclusion criteria included previous radiotherapy in the head and neck region, ongoing dental treatment or pain during chewing, documented hypersensitivity or allergy to the testing devices, and inability to comply with testing procedures. Medication intake was not considered an exclusion criterion. This article presents secondary outcomes from a previously published study, focusing on the validation of a novel test for the diagnosis of hyposalivation at rest [18].

Demographic data, medical history (including systemic diseases and medication intake), and dental history were extracted from the participants’ health records, supplemented by a brief clinical assessment of their dental health. Study participants were recruited irrespective of the type of dental care received at the University Clinics. The dental status was assessed and categorized according to the presence of natural teeth and prosthetic rehabilitation (full dentition, partially edentulous with or without removable prostheses, and fully edentulous wearing complete removable prostheses). The number of remaining teeth was recorded. Participants were asked to refrain from eating, drinking, smoking, and engaging in oral hygiene routines for a period of at least 2 h prior to the examination. The xerostomia inventory (XI), a standardized 11‐item, was employed as a summated rating scale for xerostomia symptom severity [19]. All the items were scored on a five‐point Likert scale from “never” to “very often”; therefore, the higher the total score, the more severe the xerostomia. In addition, the general question “How often does your mouth feel dry?” (never, occasionally, frequently, and always) was asked, as recommended for validation purposes. Individuals who answered “frequently” or “always” to this question were deemed to present with xerostomia for the purpose of this study [20]. The questionnaire was completed in French, the participant’s native language, with assistance available if required.

Throughout the exam, participants were seated in a relaxed posture, eyes open, with their head slightly leaning forward. Participants were asked to refrain from verbal communication to restrict the orofacial movement. To account for circadian variation in salivary flow, all salivary tests were conducted in the morning hours between 08:00 and 11:00. The tests were conducted in a pre‐established, fixed order, beginning from the measurement of unstimulated salivation to the measurement of stimulated salivary flow. The unstimulated salivary flow rate was determined by the draining method, in which saliva was allowed to flow freely from the lower lip into a preweighed tube. After 5 min, the subjects were instructed to spit out their saliva into a tube. The saliva was weighed to determine the quantity [21]. The stimulated salivary flow rate was determined with a chewing gum test, in which the subjects chewed a standardized wax gum (Saliva‐Check Buffer, GC) and saliva was gathered for a 5‐min time interval [22].

### 2.1. Statistical Analysis

Statistical analysis was performed using the IBM SPSS Statistics software (version 30, IBM Corp., Armonk, NY, USA). Continuous variables, such as age and salivary flow rate, were reported as means with standard deviations (SD) and ranges, while categorical variables, such as systemic conditions, medication intake, and smoking status, were presented in terms of frequencies and percentages. None of the continuous variables followed a Gaussian distribution according to the Kolmogorov–Smirnov test. Thus, comparisons of continuous variables were performed using the Mann–Whitney test, and correlation analysis was based on Spearman’s rho coefficient. Pearson’s chi‐square tests were used for categorical variables.

Binary logistic regression was performed to evaluate the strongest associations in a multivariate analysis. For these analyses, continuous variables were transformed into ordinal variables with 2 or 4 categories. The Wald statistic was used to evaluate the association, and backward elimination of variables was performed to highlight the strongest associations. Classification of cases by the model was used to evaluate its clinical significance. Post hoc power analysis was performed using 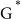
power (version 3.1.9.4). A *p*‐Value < 0.05 was considered statistically significant.

## 3. Results

A total of 116 subjects, 69 women and 47 men, were recruited with a mean age of 50.2 ± 18.7 years. All completed the entire examination and test battery. Xerostomia was identified among 14.7% of the population, and hyposalivation at rest among 21.6%. In addition, hyposalivation under stimulation occurred in 4.3% (Table 1). The average XI score was 20.5 out of 35 (Table 2). Participants with xerostomia were significantly older (62.9 years vs. 48.0 years; *p* = 0.002), and had fewer teeth (18.6 vs. 22.3, *p* = 0.03). In addition, these subjects had lower stimulated salivary flow rate (1.2 vs. 1.8, *p*  < 0.01), lower unstimulated salivary flow rate (0.2 vs. 0.3, *p* = 0.03), and their XI score was higher (32.7 vs. 18.4, *p*  < 0.01) (Table 3). Participants with hyposalivation at rest were older (60.8 vs. 47.3, *p*  < 0.01), were on a larger number of medications (1.9 vs. 0.8, *p* = 0.01), and had fewer remaining teeth (18.6 vs. 22.7, *p* = 0.03). In addition, they had significantly higher XI scores (24.9 vs. 19.3, *p*  < 0.05), and their stimulated salivary flow rate was lower (1.2 vs. 1.9, *p*  < 0.01) compared to healthy individuals (Table 4).

**Table 1:** Characteristics of the study population, prevalence of xerostomia and hyposalivation.

Condition	*N*	Percentage	Notes
Xerostomia	17	14.7	Based on single question “How often does your mouth feel dry?” Always, frequently, occasionally, or never
Hyposalivation at rest	25	21.6	Based on UWSF < 0.1 mL/min
Hyposalivation stimulated	5	4.3	Based on SWSF < 0.7 mL/min

Abbreviations: SWSF, stimulated whole salivary flow rate; UWSF, unstimulated whole salivary flow rate.

**Table 2:** XI questionnaire, correlation between unstimulated (UWSF) and stimulated salivary flow rate (SWSF).

Questions	Mean	SD	UWSF		SWSF	
			Spearman rho	*p*	Spearman rho	*p*
1. I sip liquids to aid in swallowing food	1.8	1.3	−0.046	0.625	−0.191 ^∗^	0.040
2. My mouth feels dry when eating a meal	1.4	0.8	−0.205 ^∗^	0.027	−0.160	0.085
3. I get up at night to drink	2.0	1.2	−0.066	0.480	−0.070	0.454
4. My mouth feels dry	2.1	1.2	−0.251 ^∗∗^	0.007	−0.285 ^∗∗^	0.002
5. I have difficulty in eating dry foods	1.6	1.1	−0.166	0.076	−0.201 ^∗^	0.030
6. I suck sweets or cough lozenges to relieve dry mouth	1.3	0.8	−0.282 ^∗∗^	0.002	−0.243 ^∗∗^	0.009
7. I have difficulties swallowing certain foods	1.5	1.0	−0.145	0.121	−0.174	0.061
8. The skin of my face feels dry	2.1	1.4	−0.057	0.543	−0.063	0.504
9. My eyes feel dry	2.2	1.4	−0.175	0.061	−0.147	0.115
10. My lips feel dry	2.5	1.3	−0.152	0.103	−0.253 ^∗∗^	0.006
11. The inside of my nose feels dry	2.1	1.4	−0.199 ^∗^	0.032	−0.156	0.095
XI score	20.5	7.7	−0.230 ^∗^	0.010	−0.289 ^∗∗^	<0.01

*Note:* 5‐point Likert scale from “never” = 1 to “very often” = 5.

^∗^ *p* < 0.05.

^∗∗^ *p* < 0.01.

**Table 3:** Comparison between subjects reporting xerostomia and those who do not.

Variable	No xerostomia		Xerostomia		
	*N* = 99		*N* = 17		
	Mean	SD	Mean	SD	Mann–Whitney *p*‐value
Age	48.0	17.7	62.9	19.4	<0.01
Number of teeth	22.3	9.1	18.6	9.6	0.03
UWSF	0.3	0.3	0.2	0.2	0.03
SWSF	1.8	0.7	1.2	0.6	<0.01
XI score	18.4	5.0	32.7	9.1	<0.01
Number of medications	0.9	1.5	1.8	2.5	0.12

Abbreviations: SWSF, stimulated whole salivary flow rate; UWSF, unstimulated whole salivary flow rate.

**Table 4:** Comparison between subjects presenting with hyposalivation at rest and those who do not.

Variable	No hyposalivation at rest		Hyposalivation at rest		
	*N* = 91		*N* = 25		
	Mean	SD	Mean	SD	Mann–Whitney *p*‐value
Age	47.3	17.8	60.8	18.3	<0.01
Teeth	22.7	8.8	18.6	10.0	0.03
SWSF	1.9	0.7	1.2	0.5	<0.01
XI score	19.3	5.9	24.9	11.2	0.05
Number of medications	0.8	1.4	1.9	2.3	0.02

Abbreviation: SWSF, stimulated whole salivary flow rate.

The five participants with hyposalivation under stimulation were significantly older (75.8 vs. 49.1, *p*  < 0.01), had fewer teeth (13.8 vs. 22.1, *p* = 0.03), had lower unstimulated salivary flow (0.01 vs. 0.34, *p* = 0.02) and had higher XI scores (31.0 vs. 20.0, *p* = 0.01).

Correlation analysis demonstrated that stimulated and unstimulated salivary flow were positively correlated with a Spearman rank correlation coefficient of 0.51 (*p*  < 0.01). Also, stimulated and unstimulated salivary flow were negatively correlated with age, number of drugs, and XI score (Spearman coefficient: from −0.22 to 0.39, *p*  < 0.01). Number of teeth was positively correlated with unstimulated salivary flow (Spearman coefficient of 0.23, *p* = 0.01) but not significantly correlated with stimulated salivary flow (Spearman coefficient of 0.15, *p* = 0.10) (Table 5).

**Table 5:** Spearman correlations between unstimulated (UWSF) and stimulated salivary flow (SWSF) and characteristics of the participants.

Variable	SWSF	Age	Teeth	XI score	Number of medications
UWSF	0.51	−0.39	0.23	−0.23	−0.30
*p*	<0.01	<0.01	0.01	0.01	<0.01
SWSF	—	−0.22	0.15	−0.29	−0.23
*p*	—	0.02	0.106	<0.01	0.01

## 4. XI Questionnaire

The subjects who answered “frequently” or “always” to the validation question “How often does your mouth feel dry?” answered “often” or “very often” to Question 4 (My mouth feels dry). Thus, the questionnaire was considered valid. Except for the Questions 2 and 7–9, all the items of the XI questionnaire were correlated with either unstimulated or stimulated salivary flow (Table 2).

The association between xerostomia and dentition status approached significance (*p* = 0.055), being more frequent in partially edentulous persons in one jaw wearing a removable partial denture (33.3%) and among the six fully edentulous persons wearing complete removable dental prostheses (22.2%). Also, a nearly significant association between dentition status and hyposalivation was observed (*p* = 0.07) with a greater hyposalivation rate (34.2%) among those having a more compromised dentition compared to those with a full dentition (16.0%); however, these associations disappeared when controlling for age in a logistic model since dentition status was strongly related to age, with a higher proportion of removable denture wearers among older participants.

The study identified no significant association between gender or smoking status and either of the two conditions ‐xerostomia or hyposalivation at rest. Nor was a systemic disease associated with xerostomia. Systemic disease was associated with hyposalivation at rest in univariate analysis (*p* = 0.04); however, this association disappeared when controlling for age in a logistic model since systemic disease was more prevalent as age increased.

The XI score was higher among those who had one or more systemic diseases (Mann–Whitney, *p* = 0.02) and who took at least 1 drug (Mann–Whitney, *p* = 0.02).

### 4.1. Binary Logistic Regression Analysis

Due to the nonnormal distribution of the continuous variables, these variables were transformed into ordinal or categorical variables, as follows:

Age was grouped into four categories (21–31 = 1, 32–51 = 2, 52–65 = 3, 65+ = 4).
Systemic disease (yes/no).
Number of drugs (0, 1, 2, or more).
Number of teeth (0–19 = 1, 20+ = 2).
XI score in 4 categories (11–14 = 1, 15–18 = 2, 19–24 = 3, 25+ = 4).

### 4.2. Logistic Model Predicting Xerostomia

Independent variables were selected based on the binary results. Thus, age, systemic diseases, number of drugs, number of teeth, and hyposalivation at rest were entered into the logistic model. The number of teeth was entered into the model, despite its correlation with age, as the association with the dependent variable was strongly significant. However, since participants identified with xerostomia were the same as those who answered “4 or 5” on the Likert scale of Question 4 of the XI score, the XI score was not entered in the model. Dentition status was not entered either as it was not significantly associated with xerostomia when controlling for age.

The model was highly significant (*p*  < 0.001; power 0.87) although it predicted only 85.3% of the cases correctly and never predicted a xerostomia case. A descendant selection of variables based on Wald values retained hyposalivation at rest as the best predictor of xerostomia. When excluding hyposalivation at rest from the model, age was the only variable retained (Table 6).

**Table 6:** Wald value and significance for the logistic model with xerostomia as the dependent variable.

Predictor variable	Wald	Sig.
Age	**3.322**	**0.345**
Number of medications	0.521	0.771
Systemic diseases	0.036	0.850
Hyposalivation at rest	**5.154**	**0.023**

*Note:* Bold highlights the strongest association.

### 4.3. Logistic Model Predicting Hyposalivation at Rest

In this model, the same independent variables were entered along with the XI score.

The model was also highly significant (*p*  < 0.001; power 0.60) but it predicted correctly 79.3% of the cases, and only five cases with hyposalivation at rest were predicted correctly. A stepwise backward selection of variables based on Wald values retained age only as the best predictor of hyposalivation at rest. When excluding age from the model, the number of drugs and XI score were the two variables retained (Table 7).

**Table 7:** Wald value and significance for the logistic model with hyposalivation at rest as the dependent variable.

Predictor variable	Wald	Sig.
Age	**3.538**	**0.316**
Number of medications	1.936	0.380
Systemic diseases	0.121	0.728
XI score	**5.957**	**0.114**

*Note:* Bold highlights the strongest association.

## 5. Discussion

The present study identified some key patient factors associated with xerostomia and hyposalivation in an adult group. Interestingly, 14.7% of the participants presented xerostomia, 21.6% hyposalivation at rest, and 4.3% hyposalivation under stimulation. Factors like the participant’s age and medication intake were significantly associated with xerostomia and hyposalivation at rest in the multivariate analysis. The number of remaining teeth showed associations in univariate analysis, though these relationships were largely explained by age‐related factors. In multivariate models, hyposalivation at rest emerged as the strongest predictor of xerostomia, with age as the dominant predictor when hyposalivation was excluded. For hyposalivation at rest, age was the strongest predictor, while the XI score and medication number emerged as predictors when age was excluded from the model. Notably, factors like smoking status, gender, and systemic disorders were determined to have no significant independent associations.

The prevalence rates in this study for xerostomia, hyposalivation at rest, and stimulated hyposalivation are in agreement with the rates described in the literature, although they present significant differences compared to those in some studies. An extensive systematic review estimated the worldwide prevalence of xerostomia at ~22%, with notable variability according to the studied population and the assessment methods [23]. Our lower prevalence might be explained by our exclusion of persons with a radiotherapy history, where a prevalence of hyposalivation of 78% was reported [24]. We also employed a direct questioning approach rather than a summated score cutoff approach, which has a higher probability of yielding conservative estimates. In contrast, our figure for hyposalivation at rest (21.6%) is similar to Thomson et al. [25] and collaborators’ observations of 22.1% in older South Australian populations. However, our own prevalence of stimulated hyposalivation (4.3%) was much less than the 30.5% reported in a recent meta‐analysis [6]. Perhaps this is due to methodological differences, namely, the choice of the paraffin wax test, while other studies have used other stimulants and other thresholds that may overestimate prevalence. Furthermore, reports of higher frequency typically concern patients with systemic disease or medication intake outside our population profile.

Our results confirm the multifactorial etiology of salivary dysfunction and validate a range of risk factors reported previously. Polypharmacy and age were strongly associated with xerostomia and hyposalivation—particularly at rest—in accordance with numerous studies showing the impact of medication intake and aging on salivary gland function [26, 27]. The correlation observed between advancing age and decreased salivary flow has been commonly assigned to glandular atrophy with age, comorbid conditions, and an increased rate of chronic medication intake [28]. In our population, the fact that the number of medications significantly correlated with objective and subjective measures of dryness underscores the important link between salivary dysfunction and polypharmacy. The number of remaining teeth showed associations with both xerostomia and hyposalivation in univariate analysis (*p* = 0.03); however, this relationship appeared to be largely confounded by age, as dentition status was strongly correlated with participant age, and these associations disappeared when controlling for age in multivariate models. This concurs with the existing evidence that impaired dentition can influence oral function as well as salivary stimulation [29].

Unexpectedly, sex and smoking status did not emerge as significant predictors in our study, either in multivariate or univariate models. This is contrary to some research that has indicated the female gender as a potential predictive factor for xerostomia, possibly as a result of hormonal effects [30] or that smoking may result in oral dryness [31]. However, our study indicates that these factors may have a more subtle or context‐limited impact. Also, systemic disease was correlated with resting hyposalivation on univariate analysis but lost its significance when subjected to logistic regression analysis, suggesting an indirect or mediated relation, possibly in the context of the use of medication. Last, stimulated hyposalivation was not common in our cohort (*n* = 5, 4.3%), limiting our statistical power to identify associations in multivariate models. Those affected were significantly older, had fewer teeth, lower unstimulated flow, and higher XI scores, suggesting that stimulated hyposalivation may represent a more severe manifestation of salivary dysfunction. The low prevalence could reflect the functional reserve capacity of the salivary glands, whereby stimulated salivary flow is relatively preserved in healthy individuals, or the exclusion of high‐risk groups such as radiotherapy patients from our study [32]. This emphasizes the importance of distinguishing between various states of salivary function and recognizing that both resting and stimulated flow can be influenced by different physiological and clinical mechanisms.

In the present study, the XI total score showed significant correlations with both xerostomia symptoms and objective measures of salivary dysfunction. In logistic regression analysis, hyposalivation at rest presented as the strongest predictor of xerostomia through backward elimination, with age being the only retained variable when hyposalivation was excluded from the model. For hyposalivation at rest, age was identified as the dominant predictor. When age was excluded from this model, the number of medications and XI score were the two variables retained, indicating their secondary but significant importance after accounting for age effects. Notably, the XI score could not be included in the xerostomia prediction model due to circularity, as xerostomia was defined based on a single validation question that overlapped conceptually with XI item 4. These results are in accordance with earlier reports showing a strong correlation between the XI score and reduced salivary flow rate, especially in older individuals or individuals with systemic disease and polypharmacy. Despite this, although XI is often used to measure xerostomia, it is not necessarily related to the pattern of objective assessments of salivary secretion, particularly in the context of stimulated flow. Different groups have pointed out that there is discordance between self‐reported symptoms and objective outcomes, such that psychological, neurological, and behavioral components can affect XI scores without necessarily having a direct relationship to glandular secretion [20, 29]. The XI score did not adequately predict stimulated hyposalivation in our sample, in accordance with this discrepancy. This highlights the limitation of using only symptom‐related questionnaires for diagnosis. XI is still, however, considered the “gold standard” screening test, especially when combined with objective tests [33].

An important limitation of the predictive models should be acknowledged. While both logistic regression models achieved statistical significance (*p*  < 0.001 for xerostomia model with power 0.87; *p*  < 0.001 for hyposalivation model with power 0.60), their ability to correctly classify individual cases was limited. The xerostomia model, despite achieving an 85.3% overall accuracy, failed to correctly predict any of the xerostomia cases, while the hyposalivation model correctly identified only 5 of the 25 hyposalivation cases despite 79.3% overall accuracy. This discrepancy between statistical significance and individual predictive accuracy highlights the complexity of these conditions and suggests that additional factors not captured in this study—such as mucosal sensitivity, medication‐specific effects, psychological factors, or genetic predisposition—may contribute substantially to individual risk [34, 35]. These findings underscore that clinical assessment should rely on combining multiple indicators rather than depending solely on predictive models for individual patient evaluation.

This study presents several strengths. The subjective and objective measurement of salivary function allows a clear description of xerostomia and hyposalivation. Further, the concurrent measurement of both unstimulated and stimulated salivary flow rate, together with adjustment for patient‐related variables like number of remaining teeth, age, and medication intake, yields clinically relevant findings. The use of validated tools, such as the XI, a validated and commonly used instrument, also strengthens patient‐reported outcomes. Although the sample size was computed for another outcome, the statistical power of most of the binary tests was above 0.80, supporting the robustness of the findings. However, certain limitations need to be mentioned. The use of a cross‐sectional design precludes the causal inference between risk factors and salivary dysfunction. The sample size, while adequate for primary analyses, might have limited the power to find more subtle associations in multivariate models, most particularly hyposalivation under stimulation, where there were few cases. Besides, while careful data gathering for dentition and medication was conducted, other possibly significant variables, that is, hydration status, eating habits, and psychological aspects, were not evaluated. Finally, the exclusion of radiotherapy‐treated persons, despite being essential in maintaining internal validity, could constrain the generalizability of findings to populations with an oncological medical history.

Future research might benefit from the implementation of a longitudinal study design to investigate potential causal links between patient‐related factors and hyposalivation or xerostomia. Examining the impact of changes in medication intake or dental prosthetic interventions on salivary secretion rates over time could enhance the current literature on prevention and management measures. Chewing exercises stimulate saliva secretion via the periodontal receptors but have never been tested for lasting symptom relief [36]. Additionally, extending the scope to include lifestyle variables, including levels of hydration, stress management, nutritional habits, and oral hygiene practices, may allow the identification of modifiable risk factors that are not explored in the present study. Lastly, the development of combined predictive models that merge subjective measures such as the XI with objective biomarkers might enhance early detection and risk stratification within a broader clinical practice.

## 6. Conclusion

This study highlights patient‐related factors associated with xerostomia and hyposalivation in an adult population. Age emerged as the dominant predictor for both conditions in multivariate analysis, with medication intake also showing significant independent associations. Hyposalivation at rest was the strongest predictor of xerostomia symptoms, highlighting the close relationship between objective and subjective measures of oral dryness. The XI score proved to be a significant predictor for both conditions when age was controlled for, supporting its value as a screening tool for salivary dysfunction. On the other hand, smoking and gender were not found to be significant predictors in this population. Similarly, systemic disease associations appeared to be mediated by age and medication use rather than representing independent risk factors. Assessment of these factors may be especially useful in identifying at‐risk patients and therefore strengthening preventive measures for subsequent conditions or diseases.

## Author Contributions

**Foteini Spyraki:** conceptualization, methodology, investigation, writing original draft, review and editing. **Philippe Mojon**: methodology, data curation and formal analysis, writing – review and editing. **Najla Chebib**: writing – review and editing. **Frauke Müller**: conceptualization, investigation, writing – review and editing. **Sabrina Maniewicz**: supervision, conceptualization, investigation, data curation, writing – review and editing.

## Funding

This project was entirely funded by institutional funds of the University of Geneva.

## Disclosure

All authors have read and approved the final version of the manuscript. Sabrina Maniewicz had full access to all of the data in this study and takes complete responsibility for the integrity of the data and the accuracy of the data analysis.

## Conflicts of Interest

This clinical study was conducted using institutional funding.

## Data Availability

Data will be made available upon reasonable request.
